# Economic, social, and cultural capital and ESQ in academic achievement: A comparison of Afghan and Iranian students

**DOI:** 10.3389/fpsyg.2023.1087480

**Published:** 2023-02-13

**Authors:** Reza Pishghadam, Elham Naji Meidani, Seyed Mohammad Ebrahim Momenzadeh, Saba Hasanzadeh, Mir Abdullah Miri

**Affiliations:** Department of English, Ferdowsi University of Mashhad, Mashhad, Iran

**Keywords:** cultural capital, economic capital, social capital, emo-sensory intelligence, academic achievement

## Abstract

The present study aimed to investigate the role of different types of capital, including economic, social, and cultural, as well as emo-sensory intelligence (ESI) in the academic achievement of students in the two contexts of Afghanistan and Iran. For this purpose, 317 students from both countries participated in the study. They were asked to fill out the Social and Cultural Capital Questionnaire (SCCQ) and the Emo-sensory Intelligence Questionnaire (ESI-Q). Their grade point average (GPA) was taken as the indicator of their academic achievement. The findings revealed that students’ level of cultural capital and emo-sensory quotient (ESQ) had a significantly positive role in their academic achievement (*p* < 0.05). Moreover, significant differences were found between the two contexts in terms of the level of capital, with Afghan students having significantly more cultural capital, and Iranian students having significantly higher economic capital (*p* < 0.05). Iranian students were also found to have a significantly higher level of ESQ compared to Afghan students (*p* < 0.05). Finally, the results were discussed, and implications and suggestions for further research were provided.

## 1. Introduction

Research on capital in different disciplines has grown exponentially within the past few decades. The definition of capital is no longer limited to the traditional monetary practices as put by Marx (1867), who referred to capital as a class theory related to economic conditions, money, and material possessions. In fact, different types of capital have been introduced, pioneering with Bourdieu (1986) who revolutionized the meaning of capital by introducing other forms of capital along with economic capital, i.e., cultural, social, and symbolic capital. Bourdieu (1986) did not prioritize economic capital over other kinds of capital and defined it only as financial sources. He believed that economic capital deals with social construction, which is also culturally grounded. Bourdieu’s concept of capital paved the way for other types of capital to be proposed, such as natural, physical, and human capital (Coleman, 1990), emotional capital (Nowotny, 1981), psychological capital (Luthans et al., 2004), and sensory capital (Pishghadam et al., 2019). What is common among all forms of capital is having possession of or access to resources that can eventually lead to better positions in life.

According to Blau and Duncan (1967) and Lerner et al. (2009), family socioeconomic status has a significant impact on human development in all aspects and family resources can have a significant impact on children’s academic achievement. Based on the family investment theory, children from families with higher socioeconomic status have more resources for development, such as money, which fosters better academic growth. Children from lower socioeconomic families, on the other hand, have less development capital, which impedes the improvement of academic outcomes (Conger and Donnellan, 2007). Whether or not a child can graduate, parents’ educational level and income have a great influence on their children’s access to higher education (James, 2000).

Speaking of academic achievement, Lei et al. (2018), Zhang et al. (2018), and Abid and Akhtar (2020) have shown that student learning engagement is positively correlated with it or that it can predict students’ academic performance. Although children from lower social status show greater interest in learning than children from higher social status groups, some studies have found that their academic performance is not as strong as that of higher status groups. It has been demonstrated that low academic performance among lower status children is not always due to a lack of motivation for learning. As a result, research into the social class-based influences on learning engagement is essential. This indicates that while a student’s will and effort can affect academic achievement, learning engagement does not.

Moreover, in line with emotional and sensory capital, the concept of emo-sensory quotient (ESQ), introduced by Pishghadam and Shayesteh (2017) emphasizes the essential role of senses and emotions together in experiencing and perceiving the world. Such experiences, according to these scholars, impact cognition, and thus learning. Other researchers continued this research strand and explored how emo-sensory intelligence (ESI) could impact learning (e.g., Borsipour et al., 2019; Gholami et al., 2021). Additionally, Teachman (1987), Perna and Titus (2005), Huang (2009), and Aman et al. (2019) have separately discussed the connection between academic success and social, cultural, and economic capital. However, the literature depicts no research investigating the relationship between emo-sensory quotient (ESQ) and various types of capital and their potential impact on academic achievement, simultaneously.

This study examined the two contexts of Afghanistan and Iran, which are neighboring countries located in central Asia and the Middle East, respectively. They share many cultural and historical ties, as well as the same language and religion. This study aimed to delve into the relationships among capital, ESQ and academic achievement of students in these countries and to make a comparison between the two contexts. The findings of this study can feed into a larger discussion on the bilateral and multilateral relations between the types of capitals and ESQ, contributing to the ongoing proliferation of cross-cultural research on the interdisciplinary work in educational sociology and educational psychology.

## 2. Review of literature

### 2.1. Capitals in education

According to Bourdieu (1986), cultural capital refers to social advantages and the accessibility of individuals to different cultural goods (e.g., the Internet, computers, books, and dictionaries), which fall into three categories. The first one is embodied cultural capital, which refers to appreciating and valuing cultural goods. It also encompasses attitudes, practices and cultural preferences. The second one is objectified cultural capital which involves cultural goods, such as paintings, writings, monuments, television, and radio. The last one is institutionalized cultural capital, which educational qualifications, competencies, experiences, and certificates.

In addition to cultural capital, social capital was also introduced by Bourdieu (1986), which is defined as “the aggregate of the actual or potential resources which are linked to possession of a durable network of more or less institutionalized relationships of mutual acquaintance and recognition” (p. 248). Basically, social capital refers to the possession of networks of relationships among individuals (Bourdieu, 1986). According to Putnam (2000), social capital is divided into structural and cognitive components. The structural part involves the networks and relationships that make groups and combine people, facilitating joint actions based on their expected roles (Dasgupta and Serageldin, 2000). On the other hand, the cognitive part refers to the mental processes and involves aspects like values, attitudes, trust, confidence and norms and is considered the most valuable contribution of social capital theory (Schuller et al., 2000).

Symbolic capital was the last type of capital that Bourdieu (1992) proposed. It is described as the internalized form of any type of capital and can be represented as assets of various capitals that social agents understand and appreciate (e.g., prestige and honor) (Fuller and Tian, 2006). Later, Luthans et al. (2004) introduced psychological capital which focuses on “who I am” rather than “who I know” and “what I know” (p. 46). It lies beyond social capital and refers to four positive psychological characteristics of individuals that improve their performance such as confidence, hope, optimism, and resilience.

Emotional capital was another capital proposed by Nowotny (1981) for the first time, which is a kind of social and cultural capital that deals with affective relations. She defined emotional capital as the “knowledge, contacts and relations as well as access to emotionally valued skills and assets, which hold within any social network characterized at least partly by affective ties” (p. 148). Finally, the concept of “sensory capital” was proposed by Pishghadam et al. (2019). Sensory capital refers to the amount of sensory access one has to different things, leading to various emotions and thus cognition and understanding of the world. It represents different levels of emotions made by other kinds of capital as well, such as economic, social, and cultural capital, as those with higher levels of these forms of capital may have more sensory experience and access (Pishghadam et al., 2019).

### 2.2. Economic, social, and cultural capital and academic achievement

Different researchers have investigated the impact of capital on individuals’ academic success. For instance, Pishghadam and Shakeebaee (2020) investigated the relationship capitals may have with each other and their possible influences on academic achievement. The findings revealed a significant relationship among capitals and that they play a decisive role in educational achievement, which should be considered when dealing with educational success in academic settings. Considering economic capital, studies have shown that family socioeconomic status can significantly predict students’ level of learning engagement. High socioeconomic status families can give their children better resources and educational opportunities, which is good for their development. Families with low socioeconomic status, in contrast, have relatively few resources available to their children, and the financial pressure from their families will make it challenging for the youngsters to invest in their education (Sirin, 2005; Randolph et al., 2006; Bempechat and Shernoff, 2012). Thus, minority students from disadvantaged backgrounds are more likely to be disengaged from their academic work. However, McClenney and Marti (2006) discovered that low-income students demonstrated a higher level of learning engagement than the general population. In other words, learning engagement and family background are negatively correlated. The early development of children from high and low socioeconomic-status families differs significantly, and this difference continues to affect students’ attitudes toward learning, academic completion rates, and academic achievement (Bradley and Corwyn, 2002; Waldfogel and Washbrook, 2011; Demir and Küntay, 2014).

Considering social capital, its relationship with cultural capital and academic achievement was studied by Pishghadam and Zabihi (2011). The study showed a significant relationship between social and cultural capital and the academic achievement of students. The researchers also found that literacy and cultural competence significantly predict higher educational success. Also, the relationship between social and cultural capital with academic motivation was examined by Momeni et al. (2020). Results demonstrated that the social and cultural capitals are closely related to the academic motivation that affects academic achievement. In addition, Piri et al. (2018) examined the influence of learners’ previously acquired capital on their achievement. The findings revealed that social, cultural, and emotional capitals are highly related to learner achievement and can significantly predict the achievement of learners. Also, family social network capital gives children more educational opportunities leading to higher educational achievements from the perspective of the social network. One of the major factors of social capital is the family since it is both the creator and the distributor of social capital (Dufur et al., 2013). Based on the studies, ethnic minority students’ lower academic performance is due, in part, to a lack of economic and cultural capital and, in part, a lack of resources from family and social networks (Perna and Titus, 2005; Mishra, 2020). In addition, the campus social network was found to be effective in students’ academic achievement and their post-graduation plans (Martin, 2009). In Martin’s (2009) study, students with high campus social networks and in-group participation were more likely to have high-status professions.

Considering cultural capital, Merenluoto (2009) examined different factors dealing with it, such as parent’s education level, previous school success, and the importance given to cultural activities. She explored the influence of these factors on graduate student’s educational achievement. The result revealed that cultural capital had a positive impact on students’ success and educational attainment. Parents’ educational backgrounds were found to be a positive predictor of their children’s academic success in a study on the connection between family background and children’s academic performance in Korea and Singapore (Wößmann, 2005). Additionally, studies have shown that families with higher cultural capital produce children who perform better academically (Dumais, 2002). Cultural capital is a significant family factor that affects students’ academic success. According to Cheng and Kaplowitz (2016), parents’ economic capital is closely related to cultural capital, and both of these factors influence the family’s social background and academic achievement.

In a similar vein, Crosnoe’s (2004) study revealed that students’ distant relationships with parents negatively impacted their educational achievements. This result showed the importance of family and school as main assets of providing cultural and social capital. Fan’s (2014) study also confirmed the importance of a family’s access to educational resources as having a significant impact on educational success. Hence, social and cultural capital can significantly impact learners’ success.

The absence of consistent findings about the relationship between students’ academic success or achievement and their economic, social, and cultural capital is evident by reviewing previous research. Additionally, there seems to be no comparative study on this topic, hence the current study takes two contexts into account.

### 2.3. Emo-sensory intelligence and academic achievement

Considering the undeniable and prominent role of intelligence in human cognition, different types of theories, including intelligence quotient (IQ) (Binet and Simon, 1905), emotional quotient (EQ) (Bar-On, 1988; Goleman, 1995), and sensory quotient (SQ) (Lombard, 2007) have been proposed which have always been a key factor in determining different academic achievements. The newly developed type of intelligence, which is derived from the mixture of emotional intelligence (EI) and sensory intelligence (SI) is emo-sensory intelligence (ESI) (Pishghadam and Shayesteh, 2017).

Intelligence quotient is explicitly concerned with the cognitive skills that could account for educational achievement (Binet and Simon, 1905), suggesting that people with a high degree of intelligence quotient could have better performance in the classroom, thus it can have a significant role in peoples’ life and educational success. However, Goleman (1995) argued that EQ, which is the ability to recognize, use and manage one’s emotions plays a more critical role in one’s success in life. Beyond that, Lombard (2007) maintained that SQ, which is the awareness of the primitive sensory wiring of our brain, is superior to IQ and EQ.

Emo-sensory quotient was introduced to make a reconciliation between SQ and EQ. In other words, ESQ deals with sensory emotions and the interactive nature of sense and emotion, which is technically called “emotioncy” (Pishghadam, 2015). The term emotioncy (emotion + frequency) suggests that individuals’ levels of emotions can be stimulated by different sensory inputs they receive. The concept has a six-level hierarchical matrix, namely null (0), auditory (1), visual (2), kinesthetic (3), inner (4), and arch (5). For example, if someone is not familiar with the word “avocado,” the person’s emotioncy level is zero/null. In contrast, if the person has only heard the word, the person’s emotioncy level is one. Hence, individuals’ emotioncy level toward various topics could be changed depending on the person’s level and frequency of exposure to the issues.

Having null emotioncy toward a concept makes one “avolved” toward it, whereas having auditory, visual, or kinesthetic emotioncies are considered as “exvolvement.” At the highest level of the emotioncy hierarchy is involvement, which includes inner and arch emotioncies. Therefore, when individuals use their senses to experience the world, diverse emotions are created. Individuals with a high ESQ level are excellent at identifying sensory feelings and changing their actions accordingly. Considering the interaction between emotions and senses and their influence on cognition, ESQ can play a vital role in education. Recent research by Pishghadam et al. (2022) found that ESQ is a positive predictor of academic success, along with IQ and EQ.

Considering the literature, it can be illustrated that more consideration is given to how different types of capitals are related to academic achievement, the association of capital with students’ intelligence and how exactly students’ intelligence can be related to their success are somehow overlooked. Moreover, reviewing previous studies shows a lack of consistent findings about the exact relationship between students’ types of capital and intelligence and their academic achievement. Therefore, this study aimed to fit these gaps by illuminating which types of capital and intelligence can more powerfully predict and are related to the students’ success in academic settings of Iran and Afghanistan.

Particularly, in the current study, it was hypothesized that a positive relationship exists between these three types of capital and ESQ. It was also hypothesized that these capitals, along with ESQ, have positive effects on academic achievement. Therefore, the study attempted to investigate the possible relationships between the capitals mentioned above and ESQ and the potential relations they might have with academic achievement in the two contexts of Afghanistan and Iran. To be more specific, the following hypotheses were formulated in this study.

1. There are significant relationships among economic capital, cultural capital, social capital, ESQ, and academic achievement of the participants.
2. Economic, cultural, and social capital and ESQ can significantly predict academic achievement.
3. There are significant differences between Iranian and Afghan participants in terms of their levels of social capital, cultural capital, economic capital, ESQ, and academic achievement.
4. Economic capital, cultural capital, social capital, ESQ and academic achievement are significantly correlated with one another within each country.

## 3. Methodology

### 3.1. Participants

A total of 317 individuals participated in the current study, comprising 123 Iranians (38.8%), and 194 Afghans (61.2%), 193 of whom were females (60.9%), and 124 were males (39.1%) between the ages of 14 and 40 with different socioeconomic and cultural backgrounds. Participants also had different levels of language proficiency including pre-intermediate (5%), intermediate (55.5%), and high-intermediate (39.4%). They all spoke Farsi as their mother tongue. In terms of education, the participants had different degrees such as below diploma (15.1%), diploma (14.5%), associate (8.8%), BA. (52.1%), and MA (9.5%), majoring in various fields including English majors, psychology, medical science, literature, computer science, engineering, physics and mathematics, and dentistry. It is worth mentioning that the rationale behind selecting participants from Iran and Afghanistan was, first, the feasibility of data collection from these two neighboring countries. As the researchers of this study are English language teachers and faculty members in Iranian and Afghan universities and schools, there was no difficulty gaining access to a wide range of learners in these countries. Furthermore, despite being geographically close, the two countries have a wide range of political characteristics. For instance, Afghanistan has been experiencing instability, violence and internal power struggle (Hoseini and Dideh, 2022) and, as a relatively stable country, Iran has been home to immigrant Afghans since the 1980s (Naseh et al., 2022).

### 3.2. Instrumentation

Two questionnaires were used to collect data for this study, including the Social and Cultural Capital Questionnaire (SCCQ) and the Emo-sensory Intelligence Questionnaire (ESI-Q).

#### 3.2.1. Social and Cultural Capital Questionnaire (SCCQ)

The SCCQ, developed and validated by Pishghadam et al. (2011), was first used to measure students’ social and cultural capital. The language of this scale is English, but the researchers translated it to Persian, the participants’ mother tongue, to have more accurate data. Back translation was performed to ensure the SCCQ covers the social and cultural capitals’ five-factor model, namely, social competence and social solidarity as sub-scales of social capital, and literacy, cultural competence, and extraversion as sub-scales of cultural capital. This survey contains 42 items (13 items measuring cultural capital and 29 items measuring social capital) on a Likert-type five-point scale of (1) not at all to very much (5) (see Supplementary Appendix A for sample items in English). The calculated Cronbach alpha reliability coefficient for the questionnaire in the current study was 0.92.

#### 3.2.2. Emo-sensory Intelligence Questionnaire (ESI-Q)

Emo-sensory Intelligence Questionnaire is the newly designed questionnaire that was developed and validated by Pishghadam et al. (2020). In this scale, different senses, i.e., hearing, sight, touch, movement, taste, and smell are measured by 144 five-point Likert-type questions (see Supplementary Appendix B for sample items). The emotional expressions of the participants were reduced to six primary emotions (happiness, surprise, sadness, disgust, anger, and fear). Four components of recognition (ability to identify basic emotions activated by senses), labeling (ability to articulate and mark these emotions), monitoring (ability to regulate the emotions), and management (ability to control and direct the emotions are considered in each of the items. Some items were negatively worded and then reverse-graded to avoid different types of response bias. The Cronbach alpha reliability coefficient of the scale in this study was 0.97.

### 3.3. Procedure

Both questionnaires were administered in a single form and participants answered them through Google forms. The questionnaires were shared with participants in the Telegram application and considering the fact that they were from two different countries, we decided to collect the data electronically. Participants of the study were members of multiple Telegram groups about language learning and therefore, convenient sampling was used for data collection. The purpose of the study was explained briefly at the beginning of the form. All individuals participated voluntarily and there was no need for participants to write their names. Hence, they were aware that they would be anonymous. Participants also provided demographic information (such as gender and age) and their grade point average (GPA), which was used as an indicator of academic achievement. GPA serves as the most common measure of academic achievement (York et al., 2015). Also, participants’ level of economic capital was obtained by answering questions related to their family income and living situation. The items were based on a five-point Likert scale (see Supplementary Appendix C). It took almost 30 minutes for each participant to fill out both questionnaires. To analyze the collected data, Statistical Package for Social Sciences (SPSS) and Analysis of Moment Structures (AMOS) softwares were employed. The relationships among the variables, the predictions among them, and the difference between the two contexts were obtained using Pearson product-moment correlation, Structural Equation Modeling (SEM), and *t*-tests, respectively.

## 4. Results

Pearson correlation coefficients were calculated for the variables of the study. Table 1 presents correlation results among the participants’ economic capital, cultural capital, social capital, emo-sensory quotient, and GPA. According to Table 1, it was found that GPA was positively and significantly associated with both cultural capital (*r* = 0.13, *p* < 0.05) and emo-sensory quotient (*r* = 0.17, *p* < 0.01). Significant positive relationships were also found between emo-sensory quotient and cultural capital (*r* = 0.27, *p* < 0.01), and social capital (*r* = 0.25, *p* < 0.01). In addition, social capital was correlated both with economic capital (*r* = 0.21, *p* < 0.01) and cultural capital (*r* = 0.53, *p* < 0.01). According to Cohen (1992), correlations between 0.1 and 0.3 are considered small. Therefore, the correlation of academic achievement with emo-sensory quotient and cultural capital, emo-sensory quotient with cultural and social capital, and finally social capital with economic capital have small effect sizes, and thus their strength of the relationship is considered weak.

**TABLE 1:** Correlations among economic (EC), cultural (CC) and social capitals (SC), emo-sensory quotient (ESQ), and grade point average (GPA).

	EC	CC	SC	ESQ	GPA
EC	1				
CC	0.013	1			
SC	0.212**	0.529**	1		
ESQ	-0.013	0.272**	0.246**	1	
GPA	0.023	0.129*	0.081	0.171**	1

**Correlation is significant at the 0.01 level (2-tailed). *Correlation is significant at the 0.05 level (2-tailed).

Next, to check the predictability of academic achievement in terms of emo-sensory quotient and the three capitals, SEM was run. One model was proposed for the predictive relationships of the five variables. In order to check whether the model fits the data, some fit indices were estimated (see Table 2). According to Schreiber et al. (2006), when for a model, the Chi-square (X2)/df ratio is smaller than 3, its Comparative Fit Index (CFI) and Good Fit Index (GFI) are larger than 0.90, and its Root Mean Square Error of Approximation (RMSEA) is smaller than 0.08, the model is assumed to indicate a good fit to a set of data.

**TABLE 2:** Goodness of fit indices.

	X2/df	GFI	CFI	RMSEA	SRMR
Acceptable fit	<3	>0.90	>0.90	<0.08	<0.08
Model	1.98	0.946	0.973	0.054	0.05

GFI, goodness-of-fit index; CFI, Comparative Fit Index; RMSEA, Root Mean Square Error of Approximation; SRMR, Standardized Root Mean Square Residual.

Based on Table 2, the proposed model indicated an acceptable fit (X2/df = 1.98, GFI = 0.946, CFI = 0.973, and RMSEA = 0.054). The following figure presents the schematic representation of the relationships among the five variables.

**FIGURE 1:**
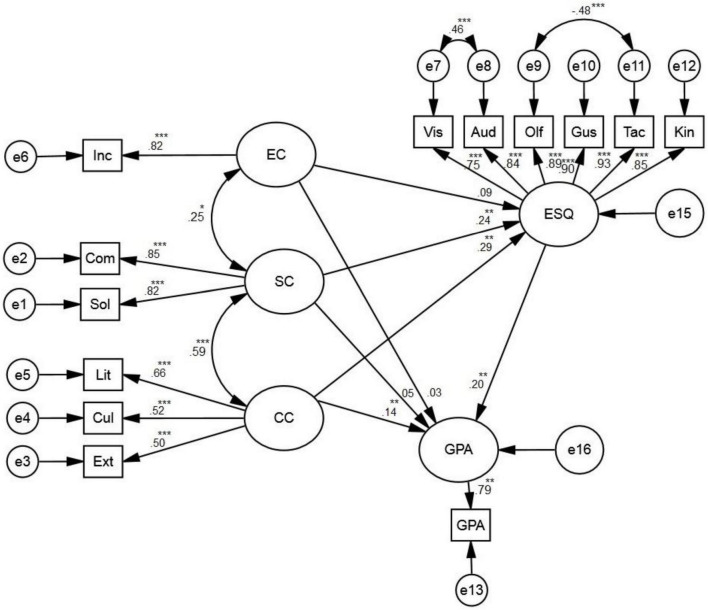
The schematic relationships among academic achievement, economic capital, cultural capital, social capital, emo-sensory quotient, and grade point average (GPA). *Significant at *p*-value = 0.05. **Significant at *p*-value = 0.01. ***Significant at *p*-value < 0.001.

As revealed in Figure 1, GPA is positively and directly predicted with the variables of emo-sensory quotient (β = 0.20, *p* < 0.01) and cultural capital (β = 0.14, *p* < 0.01). Through a bootstrap analysis with ESQ as the mediator, GPA was found to be indirectly predicted with social capital (β = 0.05, *p* < 0.05) and cultural capital (β = 0.06, *p* < 0.05). Besides, emo-sensory quotient was found to be positively and significantly predicted by social capital (β = 0.24, *p* < 0.01) and cultural capital (β = 0.29, *p* < 0.01).

To examine whether there would be a significant difference between Iranian and Afghan participants regarding their levels of social capital, cultural capital, economic capital, emo-sensory quotient, and GPA, the independent samples *t*-test was employed (see Table 4). Table 3 demonstrates the descriptive statistics of the two groups.

**TABLE 3:** Descriptive statistics of the Iranian and Afghan groups.

	Nationality	*N*	Mean	Std. deviation	Std. error mean
Social capital	Iranian	123	95.6016	16.57261	1.49430
	Afghan	194	99.0515	17.47856	1.25489
Cultural capital	Iranian	123	51.2114	8.85517	0.79844
	Afghan	194	53.6495	8.61261	0.61835
Emo-sensory quotient	Iranian	123	542.4390	84.44701	7.61433
	Afghan	194	517.4639	66.29031	4.75937
Economic capital	Iranian	123	35.12	8.996	0.811
	Afghan	194	32.27	8.518	0.612
GPA	Iranian	120	88.96	7.477	0.683
	Afghan	186	84.56	9.460	0.694

**TABLE 4:** Results of the independent samples *t*-test on the Iranian and Afghan data.

*t*-test for equality of means							
	*T*	*df*	Sig. (2-tailed)	Mean difference	Std. error difference	95% confidence interval of the difference	
						Lower	Upper
Social capital	1.75	315	0.082	-3.45	1.98	-7.33	0.43
Cultural capital	-2.43	315	0.016	-2.44	1.00	-4.41	-0.46
Emo-sensory quotient	2.78	215.18	0.016	24.97	1.00	7.28	42.67
Economic capital	2.84	315	0.005	2.85	1.00	0.85	4.85
GPA	4.52	291.36	0.000	4.40	0.97	0.85	4.85

As presented in Table 3, the mean scores of Afghan participants concerning the levels of social capital (Afghan; M = 99.05, Iranian; M = 95.60) and cultural capital (Afghan; M = 53.65, Iranian; M = 51.21) were higher than those of the Iranian participants. However, the mean scores of Iranian participants regarding levels of emo-sensory quotient (Iranian; M = 542.44, Afghan; M = 517.46), economic capital (Iranian; M = 35.12, Afghan; M = 32.27), and GPA (Iranian; M = 88.96, Afghan; M = 84.56) were higher than those of Afghan students.

Table 4 presents the results of the independent samples *t*-test for these five variables between Iranian and Afghan groups. As indicated in Table 4, there were significant differences between Iranian and Afghan participants with regards to levels of cultural capital (*t* = −2.429, *p* < 0.05), ESQ (*t* = 2.781, *p* < 0.05), economic capital (*t* = 2.844, *p* < 0.05) and GPA (*t* = 4.521, *p* < 0.05). In other words, Afghan students had significantly higher levels of cultural capital, whereas Iranian students had higher economic capital, ESQ, and GPA.

Next, to investigate the relationships among the five variables of the study within each country, two sets of correlation coefficients were calculated (see Table 5). Table 5 presents the correlations among the variables in both countries.

**TABLE 5:** Descriptive statistics and correlations among economic, cultural and social capitals, emo-sensory quotient, grade point average (GPA) for Iran and Afghanistan.

		M	SD	Social capital	Cultural capital	Economic capital	Emo-sensory quotient	GPA
Social capital	Iranian	95.60	16.57	1				
	Afghan	99.05	17.48	1				
Cultural capital	Iranian	51.21	8.85	0.49**	1			
	Afghan	53.65	8.61	0.56**	1			
Economic capital	Iranian	35.12	8.99	0.27**	-0.054	1		
	Afghan	32.27	8.52	0.21**	0.096	1		
Emo-sensory quotient	Iranian	542.44	84.45	0.33**	0.40**	0.03	1	
	Afghan	517.46	66.29	0.22**	0.22**	-0.10	1	
GPA	Iranian	88.96	7.48	0.14	0.28**	0.09	0.22*	1
	Afghan	84.56	9.46	0.10	0.11	-0.07	0.08	1

**Correlation is significant at the 0.01 level (2-tailed).

*Correlation is significant at the 0.05 level (2-tailed).

In Iran, as can be seen in Table 5, social capital had a moderate correlation with cultural capital (*r* = 0.49, *p* < 0.01) and emo-sensory quotient (*r* = 0.33, *p* < 0.01), and a weak correlation with economic capital (*r* = 0.27, *p* < 0.01). Also, emo-sensory quotient had a moderate correlation with cultural capital (*r* = 0.40, *p* < 0.01) and a weak correlation with GPA (*r* = 0.22, *p* < 0.05). Finally, GPA had weak correlations both with cultural capital (*r* = 0.28, *p* < 0.01) and emo-sensory quotient (*r* = 0.22, *p* < 0.05).

In Afghanistan, according to Table 5, social capital was weakly correlated with emo-sensory quotient (*r* = 0.22, *p* < 0.01) and economic capital (*r* = 0.21, *p* < 0.01) and largely correlated with cultural capital (*r* = 0.56, *p* < 0.01). Additionally, emo-sensory quotient had a weak correlation with cultural capital (*r* = 0.22, *p* < 0.01). However, no significant correlation was found between GPA and other variables of the study in Afghanistan.

## 5. Discussion

The first hypothesis regarding the correlations among economic, cultural, social capital, ESQ, and academic achievement was accepted. The correlational findings showed that GPA had a positive correlation with cultural capital and ESQ. In other words, a person with a high GPA could have higher cultural competence, literacy, and extraversion. For example, students with higher academic achievement could more easily welcome individual and cultural differences. This finding mirrors Merenluoto (2009)’s, Pishghadam and Zabihi’s (2011), Alanen et al.’s, (2015), Momeni et al.’s (2020), and Pishghadam and Shakeebaee’s (2020) results concerning the relationship between GPA and cultural capital. In contrast, Khodadady and Zabihi (2011) found no correlation between cultural capital and GPA. Additionally, being able to manage sensory feelings, those with higher ESQ could make better academic achievement. For instance, students with higher ESQ often experience low levels of test anxiety (Tabatabaee Farani et al., 2019), resulting in higher GPAs. The finding is also consistent with Pishghadam et al. (2020), demonstrating that minting a high GPA is associated with high ESQ scores. Furthermore, ESQ had a positive relationship with cultural capital and social capital, which indicates that a person with high ESQ tends to use more cultural goods, such as theater, music, library, museum, and is more likely to be sociable. This finding is in line with Pishghadam et al. (2019). They stated that sensory capital has a bilateral relationship with other forms of capital, such as social, economic, and cultural capital. In other words, having access to cultural and social capital leads to a more emo-sensory experience of the world and vice versa.

The second hypothesis regarding economic, cultural, social capital, and ESQ as predictors of academic achievement was also accepted. The results of SEM revealed that academic achievement is predicted positively and significantly by the variables of ESQ and cultural capital and, also positively and indirectly by social and cultural capital through the mediation of ESQ. The finding is supported by what Piri et al. (2018) found concerning the predictability of GPA through cultural capital. Moreover, this is in line with the result of Zahed-Babelan and Moenikia (2010) concerning the predictability of GPA through the components of emotional intelligence, which also complies with the findings of Williford (2000), Brackett et al. (2004), Mayer et al. (2004), and Brackett and Salovey (2006). In the same vein, Imel (2003), Márquez et al. (2006), and Berenson et al. (2008) revealed that there is a relationship between emotional intelligence and academic achievement. Also, Dufur et al. (2013), Yang (2017), and Mishra (2020) all found correlations between social capital and academic achievement.

Further, the third hypothesis regarding the differences between Iranian and Afghan participants in terms of their economic capital, cultural capital, social capital, ESQ, and academic achievement was accepted. The findings showed that the Afghan participants enjoyed higher cultural capital levels than the Iranian participants. However, with respect to ESQ, economic capital, and GPA, the Iranian participants had higher scores. Such weaker coefficients in Afghanistan might stem from the teacher-centered teaching approach in many Afghan educational settings as many Afghan teachers still use traditional teaching methods (Monib et al., 2020). In other words, teachers who have more talking times in their classes than their students and use repetitive activities are more likely not to incorporate activities that induce various senses in their teaching. Besides, with respect to economic capital, a major reason behind Afghanistan’s economic capital compared to the economic capital score in Iran is the economic difficulties Afghan people face. A recent survey by the Asia Foundation in 2019 showed that 77.7% of their respondents were concerned about the low economic situations of the families (Akseer and Rieger, 2019).

The final hypothesis regarding the correlations among economic capital, cultural capital, social capital, ESQ, and academic achievement with one another within each country was accepted as well. The study demonstrated that cultural capital in both Iran and Afghanistan is significantly correlated with social capital. Additionally, economic capital and social capital are associated with one another in both countries. Similarly, emo-sensory quotient is related to social capital and cultural capital in both countries, although the correlation coefficients are weaker for Afghanistan. Finally, GPA was found to be related to cultural capital and emo-sensory quotient in Iran, while in Afghanistan the correlations were not significant. The associations among economic, social, and cultural capital have been pointed out by Bourdieu (1992), and thus the similar results obtained in both contexts confirm the somewhat universal relationships that exist among these three forms of capital. However, the different findings obtained in the two contexts regarding academic achievement may be due to the more teacher-centered educational system of Afghanistan (Monib et al., 2020), which would undermine the role of emotions, senses, and capitals.

## 6. Conclusion

Capital and ESQ are not novel concepts. Nonetheless, the cross-cultural comparison of the findings from Iran and Afghanistan as well as their relationship and influence on academic achievement added to the novelty of the research concept. The study contributed to the body of literature on ESQ and types of capital by emphasizing that individuals with higher ESQ and cultural competence are more likely to perform better academically, as demonstrated by our data from Afghanistan and Iran, with the former having higher cultural capital and the latter having higher ESQ.

The findings of this study have some pedagogical implications. First, since the findings showed that ESQ is related to academic achievement, teachers are encouraged to create a sensory-rich environment in their classes (Shayesteh, 2019) by incorporating various sensory cues (Seyednozadi, 2021) and involving students in more sensory inputs (Hamedi, 2019). Such sensory involvement experiences could enhance long-term memory and contribute to a higher GPA (Tabatabaee Farani, 2019). Next, teachers are encouraged to prepare their curriculums based on their students’ backgrounds and interests as students with different social, cultural and economic capitals might have experienced different academic socialization processes and might have different needs. Finally, consideration of the role of social, cultural, and economic capitals, and ESQ, in academic achievement, as well as the relationships among them, would lead to a fairer learning environment, where teachers would not take learning problems as a lack of mental ability, which would leave students rather discouraged and labeled as not “intelligent” enough.

The present study confirms previous findings and adds to our understanding of the significant relationship between ESQ and different types of capitals, namely social, cultural, and economic in academic achievement. However, the results of this study should be interpreted in light of their limitations. First, since the data were collected through online questionnaires, the sample could not be considered as the representatives of all individuals, especially in Afghanistan because not everyone in Afghanistan has access to technology and the internet. Future researchers can include participants from diverse groups around the countries and collect the data using different data collection tools. Second, the study did not consider the participants’ responses in terms of gender differences; this could be an agenda for future studies. Also, prior to the collapse of the Afghan government to the Taliban, the data for this study were collected in the spring and summer of 2021. Due to the current Taliban regime in the country, the educational curriculum has been revised to reflect the ideologies of the new authorities. As a result, it is imperative to understand this critical limitation when interpreting the findings of the study. Lastly, cross-cultural studies can be done on the relationships among capitals, ESQ, and academic achievement in different regions, and contexts of the world, bringing about more insight into how these variables influence each other.

## Data availability statement

The raw data supporting the conclusions of this article will be made available by the authors, without undue reservation.

## Ethics statement

The studies involving human participants were reviewed and approved by Ferdowsi University of Mashhad. The patients/participants provided their written informed consent to participate in this study.

## Author contributions

RP conceived and designed the experiments. SM, SH, and MM performed the experiments. EN, RP, and SM analyzed the data, contributed reagents, materials, and analysis tools, and reviewed and edited the manuscript. SH, MM, SM, and EN wrote the manuscript. All authors contributed to the article and approved the submitted version.
