# Supplementary material for: Economic, social, and cultural capital and ESQ in academic achievement: A comparison of Afghan and Iranian students
Source: Front Psychol. 2023 Feb 13;14:1087480. doi: 10.3389/fpsyg.2023.1087480 (PMC9968934; doi:10.3389/fpsyg.2023.1087480)
Supplement: Supplementary file 1 [file Data_Sheet_1.docx]

**Appendices**

**Appendix A** (Sample items of the SCCQ)

I feel I have strong ties with the community.

I see my grandparents weekly.

I know a lot about literature.

I frequently buy/borrow books.

I enjoy reading (in general).

My parents know where I am, what I do.

I feel I have a strong help network for my daily activities.

**Appendix B** (Sample items of the ESI-Q)

1. I know (can distinguish) sounds that make me feel…
   sad
   surprised
   delighted
   disgusted
   enraged
   frightened
2. Expressing my feelings toward sounds that are…
   surprising is hard for me
   frightening is easy for me
   delighting is hard for me
   saddening is easy for me
   enraging is hard for me
   disgusting is easy for me
3. I can control and monitor the sorts of sounds that have ... in the past.
   frightened me
   delighted me
   enraged me
   saddened me
   surprised me
   disgusted me
4. Refraining from listening to sounds that …
   sadden me is easy for me
   delight me is hard for me
   surprise me is possible for me
   enrage me is easy for me
   disgust me is hard for me
   frighten me is hard for me
5. I know (can distinguish) images that make me feel …

Sad

Surprised

Delighted

Disgusted

Enraged

frightened

1. Expressing my feelings toward images that are …

surprising is hard for me

frightening is easy for me

delighting is hard for me

saddening is easy for me

enraging is hard for me

disgusting is easy for me

1. I can control and monitor the sorts of images that have … in the past.

frightened me

delighted me

enraged me

saddened me

surprised me

disgusted me

1. Refraining from looking at things that …

sadden me is easy for me

delight me is hard for me

surprise me is possible for me

enrage me is easy for me

disgust me is hard for me

frighten me is hard for me

**Appendix C** (Economic capital questions)

1. How do you describe your residence place?
   1. Outside the town
   2. Lower area of the town
   3. Middle area of the town
   4. Upper area of the town
2. To what extent your/family income is enough to support your living expenses?
   1. very inadequate
   2. inadequate
   3. to some extent
   4. sufficient
   5. very sufficient
3. If you divide people into 5 economic classes, in which class do you place your family?
   1. very low
   2. low
   3. medium
   4. high
   5. very high
